# Tumor perfusion enhancement by microbubbles ultrasonic cavitation reduces tumor glycolysis metabolism and alleviate tumor acidosis

**DOI:** 10.3389/fonc.2024.1424824

**Published:** 2024-07-18

**Authors:** Danxia Qiu, Yangcheng He, Yuyi Feng, Minhua Lin, Zekai Lin, Zhiyi Zhang, Ying Xiong, Zhiwen Hu, Suihong Ma, Hai Jin, Jianhua Liu

**Affiliations:** ^1^ Department of Medical Ultrasound, The Second Affiliated Hospital, School of Medicine, South China University of Technology, Guangzhou, China; ^2^ Department of Radiology, The Second Clinical College, Guangzhou Medical University, Guangzhou, China

**Keywords:** tumor acidosis, tumor glycolysis, pulsed ultrasound, contrastenhanced ultrasound, microbubbles, cavitation

## Abstract

The tumor microenvironment is increasingly acknowledged as a critical contributor to cancer progression, mediating genetic and epigenetic alterations. Beyond diverse cellular interactions from the microenvironment, physicochemical factors such as tumor acidosis also significantly affect cancer dynamics. Recent research has highlighted that tumor acidosis facilitates invasion, immune escape, metastasis, and resistance to therapies. Thus, noninvasive measurement of tumor acidity and the development of targeted interventions represent promising strategies in oncology. Techniques like contrast-enhanced ultrasound (CEUS) can effectively assess blood perfusion, while ultrasound-stimulated microbubble cavitation (USMC) has proven to enhance tumor blood perfusion. We therefore aimed to determine whether CEUS assesses tumor acidity and whether USMC treatment can modulate tumor acidity. Firstly, we tracked CEUS perfusion parameters in MCF7 tumor models and compared them with *in vivo* tumor pH recorded by pH microsensors. We found that the peak intensity and area under curve of tumor contrast-enhanced ultrasound correlated well with tumor pH. We further conducted USMC treatment on MCF7 tumor-bearing mice, tracked changes of tumor blood perfusion and tumor pH in different perfusion regions before and after the USMC treatment to assess its impact on tumor acidity and optimize therapeutic ultrasound pressure. We discovered that USMC with 1.0 Mpa significantly improved tumor blood perfusion and tumor pH. Furthermore, tumor vascular pathology and PGI2 assays indicated that improved tumor perfusion was mainly due to vasodilation rather than angiogenesis. More importantly, analysis of glycolysis-related metabolites and enzymes demonstrated USMC treatment can reduce tumor acidity by reducing tumor glycolysis. These findings support that CEUS may serve as a potential biomarker to assess tumor acidity and USMC is a promising therapeutic modality for reducing tumor acidosis.

## 1 Introduction

Solid tumors constitute a complex and heterogeneous milieu that includes aberrant vascular structures, extracellular matrix, regional hypoxia and acidosis (1). Tumor acidosis, a physicochemical hallmark of the tumor microenvironment (TME), is associated with malignant progression and even challenges for conventional cancer therapies (2). Cancer cells undergo metabolic shift toward glycolysis regardless of oxygen availability (Warburg effect) due to the aberrant vascular structure and poor blood perfusion in TME are unable to meet the highly proliferative need of tumor cells (3). This metabolic shift gives them an edge in proliferation even without adequate oxygen (4–6). The increased glycolysis generates acidic by-products (lactic acid and protons), thereby inducing tumor acidosis with a typical extracellular pH(pHe) range between 6.0 and 7.0 (7). Increased glycolytic rate and acidic waste products are linked with tumor aggressiveness, cancer treatment resistance (8–10), and metastasis (11–13). Moreover, tumor acidosis bolsters immunotherapy resistance by promoting an immunosuppressive TME (14). High glycolytic activity in tumor cells leads to tumor cell-mediated glucose restriction, dampens the ability of T cells to produce IFN-γ, suppresses anti-tumor effector functions of Th1CD4^+^ T cells by limiting the Ca2^+^-NFAT signaling pathway (15, 16).

These discoveries have fostered the need to noninvasively map tumor pHe and modify tumor acidosis through strategies such as buffer therapy by neutralizing acidic components and proton-pump inhibitors(PPIs) by blocking the export of protons (17, 18). Despite the potential of antiacids, challenges in dosage control and side effects limit their application. The colon cancer model with PPI administration showed enhanced tumor growth and liver metastasis due to gastrin and YAP activation, suggesting that PPI use in colorectal cancer patients might create a risk of cancer promotion (19). Ultrasound-stimulated microbubble cavitation (USMC) is a therapeutic ultrasound strategy that employs pulsed ultrasound to stimulate mechanical oscillations in circulating microbubbles to form beneficial bio-effects (20). Our previous research has demonstrated that USMC can enhance tumor regional blood flow perfusion and reduce tumor hypoxia when the ultrasonic amplitude is low. Therefore, USMC may become one of the breakthroughs to alleviate tumor acidosis. Previous studies have discovered that USMC could enhance blood perfusion in skeletal muscle and myocardium, likely through shear-dependent ATP increases and the purinergic pathway (21, 22). Yet the aberrant tumor vascular structures necessitate distinct considerations from normal tissue vasculature. Additionally, inappropriate acoustic parameters, such as high-pressure amplitude, can damage tumor microvasculature, leading to thrombus formation and cell necrosis (23, 24). Taken together, optimizing acoustic parameters for USMC treatment to enhance tumor blood perfusion is perhaps a critical step toward reducing tumor acidosis effectively.

Noninvasive techniques for assessing tumor pHe such as magnetic resonance spectroscopy (MRS), positron emission tomography (PET), optical imaging (OI), and photoacoustic imaging (PAI) (25–27), are limited by availability (28) or penetration depth (29), constraining their clinical use. Contrast-enhanced ultrasound (CEUS) is a relatively new technique that has been validated to noninvasive visualization and quantification of tumor perfusion (30). Our previous findings also indicated a strong correlation between CEUS-related parameters and tumor oxygen pressure. However, a comprehensive evaluation of tumor acidity by CEUS is currently lacking.

Based on the above consideration, we therefore tracked CEUS perfusion parameters (AUC, PI) in MCF7 tumor models and compared them with *in vivo* tumor pHe recorded by pH microsensor. The result showed a positive association between CEUS parameters and tumor pHe. We then conducted the USMC treatment on MCF7 tumor-bearing mice, tracked changes of tumor blood perfusion and tumor pHe before and after the USMC treatment under various acoustic pressures to evaluate its influence on TME acidity. Furthermore, tumor vessel immunofluorescence and tumor collagen fiber data were spatially registered to CEUS data for analysis of the spatial correspondence between these metrics after USMC treatment. Glycolysis-related metabolites, or enzymes, were performed to further elucidate the underlying mechanisms.

## 2 Materials and methods

### 2.1 Xenograft tumor model

All animal procedures were conducted according to the approved procedures of the Laboratory Animal Ethics Committee of the South China University of Technology (reference number 20210113). Tumor cell line MCF7 was bought from Procell company. Female BALB/c nude (4-6 weeks; 20–25 g; Guangdong Medical Laboratory Animal Center) were inoculated subcutaneously on the left flank with tumor cells (1×10^6^ cells/mouse) resuspended in 100μL DMEM basal medium. US imaging was performed when the subcutaneous tumor reached around 5 mm–1 cm in diameter. Animals with tumor ulcerations or ≥2,000 mm^3^ were euthanized and excluded from the study.

### 2.2 Ultrasound imaging

B-mode and CEUS imaging were obtained from Philips EPIQ7 (Philips Healthcare, Seattle, WA, USA, eL18-4probe, frequency 4–18 MHz). Mice were anesthetized with 1% pentobarbital and placed on a heating pad with positioned supine. B-mode images were acquired for axial planes covering the maximum tumor cross sections to record the size of tumor. After B-mode imaging, CEUS imaging was performed following a bolus injection of 20μL microbubble contrast agent (perfluoropropane, 2μm in size, Ultrasound Department of the Second Affiliated Hospital of Army Medical University) via the lateral tail vein, followed by an immediate 0.1ml saline flush. During CEUS, the depth, gain, and other settings all remained constant. CEUS videos were recorded for 1min23s starting just before the microbubbles injection. Regional contrast intensity within the tumor was visually evaluated to identify relative hypoperfusion and hyperperfusion ROIs for subsequent pH measurements.

### 2.3 Tumor pHe measurement

Calibration was conducted every time a new needle-type pH microsensor (PreSens Precision Sensing GmbH, Regensberg, Germany) was employed. The pH microsensor was securely attached to a micromanipulator (TOW TECH, Shanghai) and carefully inserted into the subcutaneous tumor tissue under ultrasound guidance, ensuring placement at the maximum tumor cross-section. Subsequently, the insertion continued until reaching the center of the hypoperfusion or hyperperfusion region (as observed in CEUS images). Once inserted to the target region, the pH microsensors were extended outside the needle housing by 1 mm to expose the fiber optic tip (microsensor). pH measurements were taken in 5s intervals after the pH recording was stabilized, which typically occurred 3 mins after extending the pH microsensor. For correlation analysis between CEUS blood perfusion parameters [Peak intensity (PI) and area under the curve (AUC)] and tumor acidity, tumor pHe was measured following CEUS imaging. To assess the impact of USMC on tumor acidity, tumor pHe was recorded both before and after the USMC treatment.

### 2.4 US images processing

Dynamic CEUS imaging was analyzed using QLAB software (Philips Healthcare). Hyperperfusion region of tumor (a 2.5×2.5mm rectangular region of interest (ROI) centered on the pH measurement point) and hypoperfusion region of tumor (same as hyperperfusion region) were manually delineated. QLAB then automatically generated a TIC curve for contrast intensity of ROIs, including the PI and AUC data. PI is defined as the peak value of the TIC minus the initial background value, and AUC represents the area under the TIC.

### 2.5 Ultrasound-stimulated microbubble cavitation treatment protocols

To explore the bioeffects induced by USMC with different acoustic pressures, 40 tumor-bearing mice were randomly divided into 5 groups: 0.5Mpa, 0.8Mpa, 1.0Mpa, 1.5Mpa, and the control, with 8 mice per group. During USMC treatment, the custom transducer (Guangzhou Doppler Electronic Technologies Co., Ltd.) was placed in contact with the tumor surface, separated with a 2-cm-thick acoustic pad, while the pulsed therapeutic ultrasound device (Shenzhen Wilde Medical Electronics Co., Ltd., models dct-700III) was turned on for 10 minutes. Apart from the acoustic pressure, other parameters were constant, including the frequency of 1 MHz, the pulse repetition frequency of 40 Hz, the duty cycle of 0.2%, and the pulse emission/gap time of 9s/3s. 0.03 ml microbubble was diluted with 0.3 ml saline and slowly injected through the tail vein during USMC procedure. Tumor blood perfusion was assessed by CEUS before and after USMC treatment. Tumor pHe was measured before and after USMC treatment.

### 2.6 lactic acid and lactate dehydrogenase concentration measurements

Mice were sacrificed, and tumor tissues were collected 30mins after the USMC treatment. The tumor lactic acid and lactate dehydrogenase (LDHA) concentrations were measured according to the instructions of the lactic acid test kit (A019-2-1, Nanjing Jiancheng Bioengineering Institute) and the lactate dehydrogenase test kit (A020-1-2, Nanjing Jiancheng Bioengineering Institute).

### 2.7 HE staining

Mice were sacrificed 30mins after the USMC treatment. The tumor tissue was fixed in formalin, embedded in paraffin, sectioned serially, and stained using H&E. HE staining was used to observe the tumor micro-vessels and the surrounding changes.

### 2.8 Immunofluorescence staining

The whole tumor tissue from MCF7 tumor-bearing mice was extracted and cut in half following the pH microsensor needle path to match the US imaging plane. Tumor tissues were then fixed with 4% paraformaldehyde, followed by blocking with 5% BSA for 30 min. Then sections were incubated with anti-CD31 antibody (1:1000, Servicebio) overnight in the dark at 4°C. After washing with PBS three times, the sections were incubated with secondary antibodies Cy3-conjugated anti-IgG (1:500, Servicebio) for 1 hour at 37°C. Following a final washing step, the nuclei were stained with DAPI. Finally, sections were mounted and imaged using fluorescence microscope (NIKON Eclipse ci).

### 2.9 Masson’s trichrome staining

Tumor tissue removal process is consistent with immunofluorescence staining. Tumor sections collected were then subjected to Masson’s trichrome staining to assess the effect of the USMC treatments on collagen fibers in each group.

### 2.10 Pathological staining sections registration and analyses

Immunofluorescence and Masson’s trichrome staining images were registered with ultrasound images through translation, rotation, and scaling adjustments (BigWarp, Fiji plugin). Subsequently, hyperperfused and hypoperfused ROIs, consistent with those identified in CEUS images, were manually delineated. The collagen area and microvessle density (MVD) in different tumor perfusion ROIs were then calculated (Fiji).

### 2.11 ELISA

Tumor tissue removal timing is consistent as above. The levels of HIF-1a and PGI2 in tumors were detected using anti-HIF-1a antibody ELISA Kit (SU-B10516, Shanghai Enzyme-Linked Biotechnology) and anti-PGI2 antibody ELISA Kit (E-EL-0022, Elabscience Biotechnology) according to the manufacturer’s recommended procedures.

### 2.12 Statistical analysis

Results are presented as means± the standard deviation (SD) or median. The Shapiro-Wilk test was used to determine whether the scale variable distributions were normal. Multi-group comparisons were tested for significant differences using one-way ANOVA followed by Tukey’s or Kruskal-Wallis H followed by Nemenyi test. Group comparisons before and after USMC treatment were performed using the two-tailed paired t-test or Wilcoxon test. To test for linear relations between different CEUS parameters and tumor pHe, linear regression analyses were performed. Statistical significance was set at *p* < 0.05. Statistical analysis was performed using R software (version 4.3.3). The R language “ggplot2” package was used for plotting.

## 3 Result

### 3.1 Contrast Enhanced Ultrasound detection of the MCF7 breast tumor

**Figure 1:**
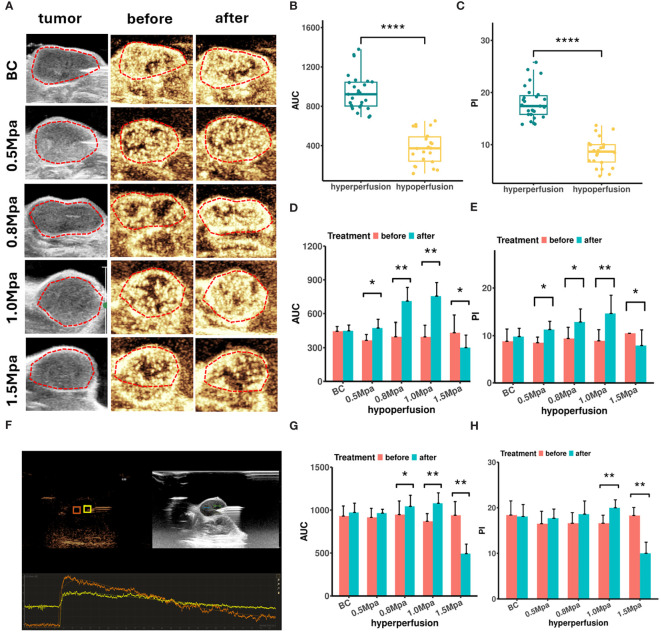
**(A)** Peak intensity CEUS images of MCF7 tumor before and after USMC treatment. **(B, C)** The AUC **(B)** and PI **(C)** values of CEUS in different blood perfusion regions before USMC treatment. **(D, E)** The AUC **(D)** and PI **(E)** values of hypoperfusion ROIs before and after treatment. **(F)** The CEUS quantitative analysis system measured the PI and AUC at the hypoperfusion ROI and hyperperfusion ROI. **(G, H)** The AUC **(G)** and PI **(H)** values of hyperperfusion ROIs before and after treatment. *P < 0.05, **P < 0.01, ***P < 0.001.

B-mode ultrasound images showed that the average length of the tumors was 10.36 ± 0.65 mm, and the average width was 6.13 ± 0.99 mm. Color Doppler flow imaging (CDFI) showed limited blood flow within MCF7 lesions, with only sparse blood flow observed. CEUS examination showed a “fast in and fast out” pattern in MCF7 lesions (**Figure 1F**). Tumor rims initially exhibited heterogeneous enhancement, followed by rapid contrast agent perfusion into the interior area, then reaching peak intensity. Contrast agent (microbubble) washed out at around 1min30s. We recorded a total of 26 tumor lesions (52 ROIs) with successful CEUS data for correlation analysis, which had a mean PI of 13.7 ± 2.3 dB and a mean AUC of 684 ± 102 dB·sec. Therefore, ROIs with AUC < 686 dB·sec and PI < 13.7 dB were categorized as hypoperfused, otherwise as hyperperfused (**Figures 1B, C**).

CEUS was also performed before and immediately after treatment to evaluate how the cavitation effects influence tumor blood perfusion. All tumors demonstrated a similar contrast enhancement and no statistical difference in PI or AUC before treatment. CEUS data showed that the USMC treatment enhanced tumor blood perfusion visually in the 0.8 Mpa and1.0 Mpa group in varying degrees, no matter in the hyperperfusion or hypoperfusion ROIs (**Figures 1A, D, E, G, H**). The USMC 1.0 Mpa treatment improved tumor blood perfusion most after the treatment, particularly in hypoperfusion ROIs, showing an increase of 5.70 ± 1.42 dB of PI and 358.43 ± 116. 32 dB·sec of AUC. By comparison, hyperperfusion ROIs showed smaller improvement in PI and AUC. An increase in tumor blood perfusion was also detected in the 0.5 Mpa group in hypo-ROIs, but no significant differences were found in hyperperfusion regions. However, the USMC 1.5 Mpa treatment significantly reduced blood perfusion, no matter in the largest tumor plane, hyperperfusion or hypoperfusion regions. (**Figures 1A, D, E, G, H**).

### 3.2 AUC and PI were strong predictors of tumor acidity

**Figure 2:**
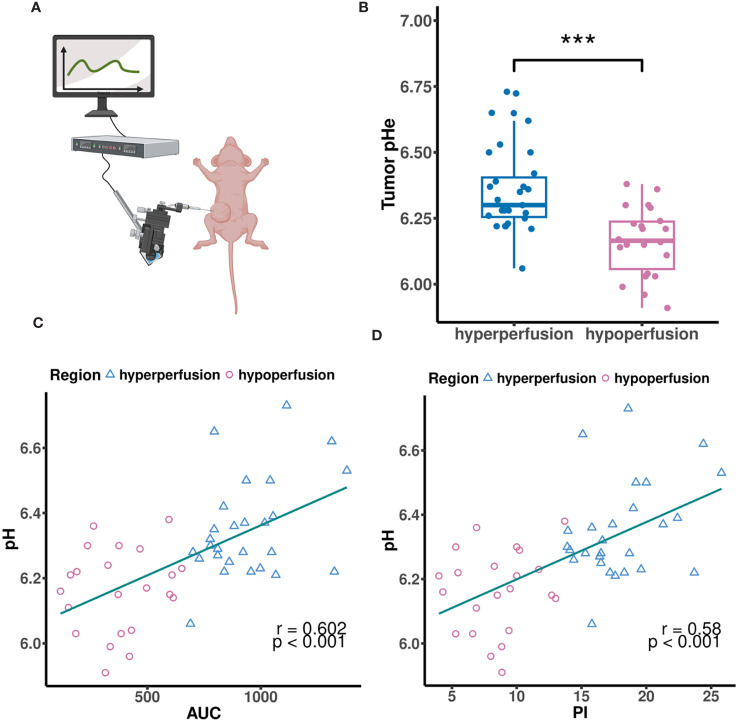
**(A)** The MCF7 tumor pHe was measured by a tissue pH microsensor. **(B)** The MCF7 tumor pHe in different perfusion regions. **(C, D)** Scatter plots showing correlations between tumor pHe and AUC **(C)**, PI **(D)**. **(A)** created with BioRender.com.

We measured tumor pHe using a pH microsensor, assuming it as the “gold standard” (**Figure 2A**). For correlation analysis between CEUS parameters and tumor pHe, we only conducted a single-time measurement of tumor pHe for each ROI using the microsensor, because the microsensor was mechanically fragile and frequently broke during use. Additionally, the fluorescent coating was easy to scrub and damage after repeated measurements. MCF7 tumor tissues all exhibited acidity, ranging from pH 5.91 to 6.73, with an average pH of 6.35. Hyperperfusion regions showed higher pHe compared to hypoperfusion regions (*p* < 0.001, **Figure 2B**). To further illustrate if CEUS perfusion parameters could be used to predict the tumor pHe, we performed correlation analysis between tumor pHe and CEUS data in both hypo-and hyperperfusion ROI. The result showed that AUC and PI were all positively correlated with tumor pH (r = 0.602, 0.58, *P*< 0.001, **Figures 2C, D**). To improve the prediction efficiency, we developed a multiple linear regression model based on the above independent influencing components (AUC, PI). However, due to notable collinearity between AUC and PI, the final predictive model excluded PI as a coefficient.

### 3.3 Tumor pHe changes after microbubbles ultrasonic cavitation

**Figure 3:**
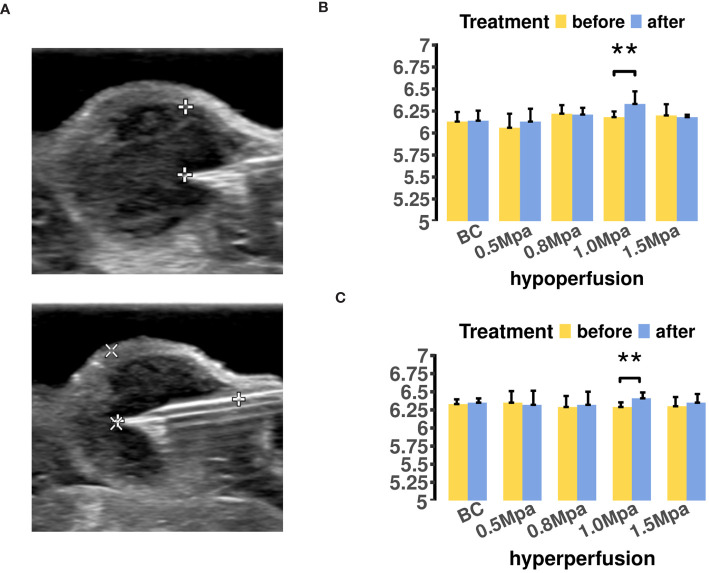
**(A)** The MCF7 tumor pHe measurement in different region was located by B mode ultrasound. **(B)** The tumor pHe values of hypoperfusion ROIs before and after USMC treatment. **(C)** The tumor pHe values of hyperperfusion ROIs before and after USMC treatment.

We conducted a single-time measurement of tumor pHe for each ROI before and after USMC treatment (**Figure 3A**). The results indicated increases in tumor pHe across different perfusion areas after 1.0MPa treatment (**Figures 3B, C**). Surprisingly, the tumor pHe didn’t show a decrease after USMC1.5Mpa treatment while a decrease in tumor blood perfusion was observed in the USMC1.5Mpa group. Meanwhile, the tumor pHe in the remaining groups showed no significant change after the USMC treatment.

### 3.4 The effects of USMC treatment on tumor microvascular related histology and molecular changes

**Figure 4:**
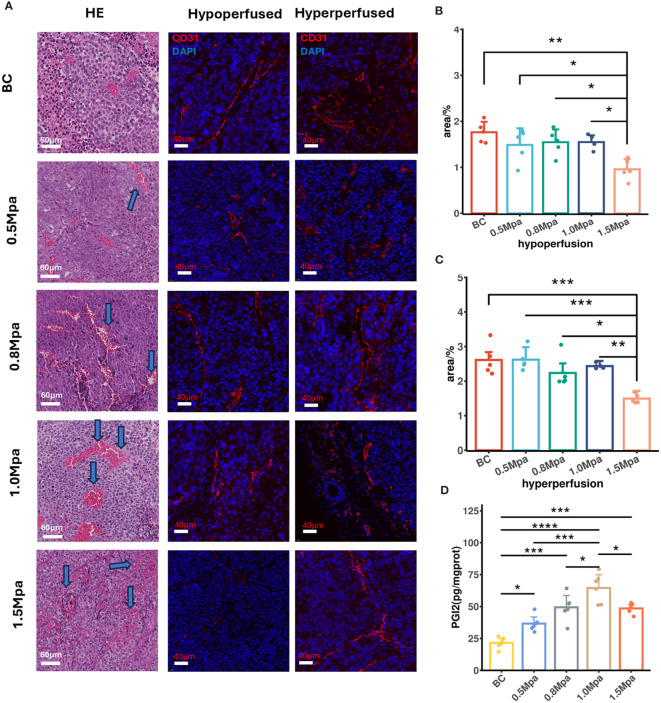
**(A)** Vessel histological findings by HE and CD31 immunofluorescence (scale bar: 60 μm and 40 μm). Arrows in the USMC 0.5, 0.8 and 1.0Mpa indicated vasodilation of microvessel. Arrows in the USMC 1.5Mpa indicated disruption of microvessel. **(B, C)** The tumor microvascular area of different perfusion ROIs after USMC treatment. **(D)** The tumor PGI2 level in each group after USMC treatment. *P < 0.05, **P < 0.01, ***P < 0.001.

To analyze CEUS perfusion parameters and their corresponding histological changes after USMC treatment, we therefore performed registration of all immunofluorescence and Masson staining images with CEUS data. This ensured that the analysis of pathological data was performed in the same region as the ROIs where we measured CEUS parameters. H&E staining and CD31 immunofluorescence were utilized to assess the tumor vasculature after USMC treatment. Results from HE staining showed that the pathological changes in the USMC 0.5 Mpa and 0.8 Mpa groups were similar: mild vasodilation of tumor microvessels was found under light microscopy, with a clear structure, complete and continuous vessel walls, and no clear red blood cell escape extravasation observed (**Figure 4A**). Notably, in the 1.0 Mpa groups, tumor microvessel dilation and congestion were most obvious, and a small amount of red blood cell leakage was scattered around blood vessels (**Figure 4A**). Meanwhile, despite visible vasodilation in the 1.5 Mpa group, it was primarily characterized by extensive microvascular damage and hemorrhage within tumor tissues (**Figure 4A**). Similarity, the USMC1.5 Mpa treatment reduced MVD in both hypo- and hyperperfusion ROIs, attributing to its microvasculature disruption (**Figures 4A–C**). Interestingly, no obvious CD31 expression increase was shown in the USMC0.5Mpa, 0.8Mpa, 1.0Mpa groups, suggesting the enhanced blood perfusion was due to vasodilation rather than angiogenesis (**Figures 4B, C**). To further explore the mechanism of tumor blood perfusion promotion after USMC, we analyzed the tumor PGI2 level after USMC treatment. We observed a significant elevation in PGI2 levels in the USMC 0.5 Mpa, 0.8 Mpa, 1.0 Mpa, with the most substantial increase noted in the 1.0Mpa group (**Figure 4D**). Surprisingly, PGI2 level in USMC 1.5Mpa was also significantly increased, while the CEUS showed that USMC 1.5Mpa significantly reduced blood perfusion (**Figure 4D**). Together, H&E staining, CD31 immunofluorescence and PGI2 assay results all demonstrated that USMC could modulate tumor perfusion and induced different histological vessel changes when applying different acoustic pressure treatments.

### 3.5 Masson’s trichrome staining

**Figure 5:**
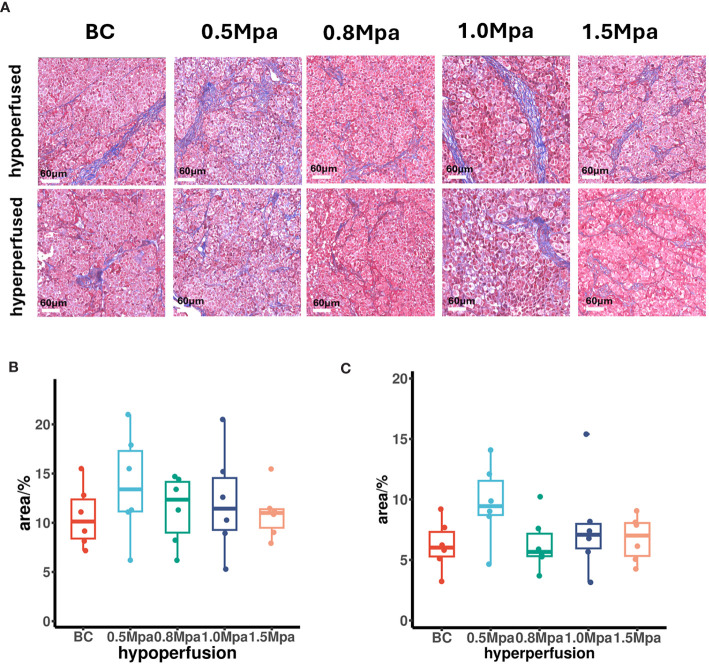
**(A)** Collagenous fiber findings by Masson’s trichrome staining (scale bar: 60 μm). **(B, C)** The tumor collagenous fiber area in different perfusion ROIs after USMC treatment.

At steady state, H^+^ venting flux rate is constrained by the tissue’s capacity to remove acid (31). Collagen fibers are the primary component of the tumor ECM. We therefore performed Masson staining on the largest section of the tumor to evaluate the effect of USMC treatment on tumor collagen fibers. In Masson’s trichrome stained-sections, collagen fibers appear blue while the blood cells and cytoplasm are red. In both hypo- or hyperperfusion regions, there was no significant difference in the collagen fiber density among all groups (**Figure 5**), suggesting that the USMC parameters we currently apply do not influence tumor tissue collagen fiber density.

### 3.6 The effect of USMC on tumor glycolytic metabolism

**Figure 6:**
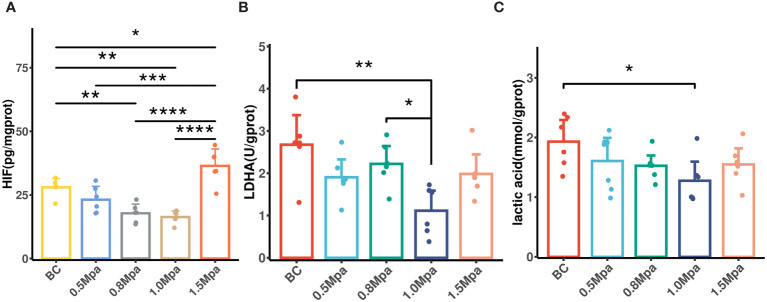
**(A)** The tumor HIF-1a level in each group after USMC treatment. **(B)** The tumor LDHA level in each group after USMC treatment. **(C)** The tumor lactic acid concentration level in each group after USMC treatment. *P < 0.05, **P < 0.01, ***P < 0.001.

Tumor blood perfusion promotion may reduce hypoxia, which in turn impacts tumor glycolysis metabolism. Therefore, we measured the tumor HIF-1α, lactate dehydrogenase and lactic acid concentrations to evaluate the effect of USMC on tumor glycolytic metabolism. The HIF-1α level in the USMC 1.0 Mpa and 0.8 Mpa groups were clearly lower than that in the control group (**Figure 6A**), which proved that the USMC with 1.0 Mpa and 0.8 Mpa treatment could effectively alleviate tumor hypoxia. However, the USMC 1.5Mpa treatment induced an increase in HIF-1α level (*p*<0.05, **Figure 6A**). There was no significant difference in HIF-1α level in the remaining groups. Recent research found that downregulation of HIF-1a can lead to a decrease in tumor glycolytic metabolism related pathways (32). We then performed tumor lactate dehydrogenase assays after USMC treatment. It was found that the tumor lactate dehydrogenase level in the USMC 1.0Mpa group was clearly lower compared with that in the control group (*p* = 0.0012, **Figure 6B**), indicating that the USMC with 1.0 Mpa treatment could effectively alleviate tumor lactic acid production. Meanwhile, there was no difference in lactate dehydrogenase in the remaining groups. Lactic acid concentration assay was conducted on tumor tissues to assess the impact of USMC treatment on tumor lactic acid levels. Consistent with the changes in tumor pHe and lactate dehydrogenase level observed after USMC treatment, the 1.0 MPa USMC treatment group exhibited a significantly reduced tumor lactic acid concentration relative to the control group (*p* < 0.05, **Figure 6C**). Surprisingly, the lactic acid level of the 1.5 Mpa group after treatment also showed no significant difference compared with the control group. Meanwhile, the tumor lactic acid level after treatment in the 0.5 Mpa and 0.8 Mpa groups also did not significantly differ from the control group.

## 4 Discussion

Tumor acidosis is essentially the result of metabolic reprogramming and poor vascular perfusion. As the tumor expands, the diffusion of nutrients and O_2_ between the blood supply and the cancer cells becomes inefficient, shifting cell metabolism towards aerobic glycolysis and resulting in lactate and H^+^ buildup in the intracellular space (11). Cancer cells can also derive energy from non-glucose-dependent pathways such as glutaminolysis in the presence of oxygen (33, 34), producing lactic acid and contributing to tumor acidosis. Additionally, CO_2_ produced by the respiration of oxidative cancer cells, converting to H_2_CO_3_ (35), serves as another significant contributor to intracellular accumulation of H^+^. To counteract the influences of intracellular acidification on cancer cell function, cancer cells are equipped with well-developed acid extruders (e.g., Na^+^/HCO3^−^-cotransporters, Na^+^/H^+^-exchangers, H^+^-ATPases and MCTs) that remove acid equivalents and lactate from the cytosol, thus increasing extracellular acidity. Additionally, the extracellular matrix (ECM) exacerbates this by compressing blood vessels and impairing lymphatic drainage, hindering acidic waste removal and increasing interstitial acidity (31, 36).

Tumor acidosis provides an environmental selection pressure that promotes oncogenic mutations, angiogenesis, metastatic potential. Lactate can upregulate VEGF receptors, triggering angiogenesis independently of hypoxia (37). Low extracellular pH also contributes to therapeutic resistance. The acid-outside pH gradient generated between intra- and extracellular space affects the distribution and uptake of select weak base chemotherapeutic drugs, resulting in drug resistance (38). Further, tumor acidification can impair the function of anti-tumor effector cells (such as T cells and NK cells) (39, 40), while facilitating the recruitment and activation of immunosuppressive cells (such as MDSCs and Tregs) (14, 41), thereby promoting immune evasion in tumors. Previous study suggest that T cells subjected to acidic conditions demonstrate increased activation thresholds, necessitating costimulatory signals such as CD28 agonism for complete activation (42). In addition, lactate itself functions as a signaling molecule, an antioxidant, and enhances immune escape, highlighting its complex role in the TME (43).

CEUS improves the resolution and sensitivity of blood echoes by intravenously injecting ultrasound contrast agent (microbubbles). This dynamic imaging modality is widely recognized for observing tissue microvascular perfusion (44), and it may have the potential to detect tumor acidity. Distinct from diagnostic ultrasound, therapeutic ultrasound with microbubbles can induce biophysical effects such as enhancing blood perfusion under appropriate acoustic settings (20, 45, 46). Furthermore, therapeutic ultrasound also has the potential to remodel extracellular matrix (ECM), expanding extracellular spaces (ECS) and increasing hydraulic conductivity (47, 48). This modification may facilitate the removal of acidic waste. Given the context, we applied CEUS to acquire tumor perfusion data (AUC and PI) to evaluate tumor acidity and found a positive correlation between CEUS parameters and tumor acidity. Then we applied USMC treatment to MCF7 tumor-bearing mice, tracking changes in CEUS perfusion parameters and tumor pHe before and after USMC treatment. Our aim was to noninvasively measure tumor acidity using ultrasound, explore the effectiveness of USMC in reducing TME acidosis, and identify the best acoustic pressure for reducing tumor acidity.

Correlation analysis showed that tumor pHe correlates well with PI and AUC, suggesting that tumor acidity is positively related to tumor vascular perfusion. Mechanically, tumor cells in the hypoperfusion region experience oxygen scarcity and in turn depend heavily on glycolysis, which then aggravates tumor acidity, and vice versa (49). However, our result showed that tumor pHe remains acidic even when AUC exceeds 1000dB·sec, supporting the accepted view that tumor cells favor aerobic glycolysis even under normoxic conditions (50). This preference enables tumor cells to withstand conditions of metabolic stress, notably during metastasis (51).

Compared with other medical imaging techniques, CEUS is distinguished by its accessibility, ease of use, and cost-effectiveness. Magnetic Resonance Spectroscopy (MRS) can also track changes in tumor pHe noninvasively, but not widely available. MRS is further limited by longer imaging times and higher costs (25, 52). The microbubbles used in CEUS range in diameter from 1 to 10 μm, similar to the size of red blood cells, preventing them from penetrating the vascular endothelium and making them pure blood pool agents (53). After circulating in the bloodstream for several minutes, the microbubbles dissolve and the gas exhaled through the lungs instead of renal route (54). Thus, CEUS has fewer side effects and contraindications than MR contrast agents, making it a suitable alternative for preliminary assessment of tumor acidosis, especially when MR contrast is not an option.

After confirming that tumor blood perfusion can indicate tumor acidity and CEUS can act as biomarkers for acidosis-targeted therapies, USMC treatment was administered to mice bearing MCF-7 tumors. Notably, USMC 1.5 Mpa significantly decreased tumor perfusion and vascular density, which was possibly due to the disruption of tumor microvascularity by high pressure amplitude ultrasound combined with microbubbles. Under high-amplitude ultrasound irradiation, microbubbles rapidly expand and contract, ultimately bursting and collapsing (inertial cavitation). The energy released from this collapse is subsequently converted to irreversible cell or tissue damage, causing necrosis and apoptosis of cancer cells, increasing vessel permeability, microvascular rupture, and thrombosis (55–57). On the contrary, low acoustic pressure (0.5-1.0MPa) USMC significantly improves blood perfusion across both hypo- and hyper-vascular regions, most notably at 1.0 MPa. This enhancement likely results from the mechanical stimulation of the vascular wall, which triggers an inflammatory response that leads to vasodilation and augmented blood flow. It has been reported that microbubble stable cavitation begins at a peak negative pressure of approximately 0.25 MPa, generating jets and microstreams from bubble oscillations that produce high intravascular shear stress (58). This stress increases shear-dependent ATP and ATP release-mediated calcium wave propagation to activate calcium-dependent purinergic signaling pathways (59). Purinergic signaling pathways are vital for vasodilation and enhancing perfusion by activating endothelial nitric oxide synthase (eNOS) and prostaglandin (PG) production (60). PGI2, a vasodilator of the PG family, is known to significantly dilate tumor microvessels (61). ELISA analysis showed that PGI2 levels increased after USMC at 0.5-1.5 MPa. This aligns with our HE staining observations: USMC at 0.5-1.5 MPa caused varying degrees of vascular dilation. Despite more significant microvascular damage and hemorrhage at 1.5 MPa, some tumor vessels still exhibited dilation, consistent with elevated PGI2 levels at this pressure.

The absence of changes of MVD in USMC 0.5, 0.8Mpa, 1.0Mpa, as evidenced by CD31 immunofluorescent staining, confirms that the increase in blood perfusion is not mediated by angiogenesis. It also implies that ultrasonically enhanced blood flow may not promote tumor growth and metastasis. Previous study also suggested that therapeutic ultrasound with microbubbles induced perfusion enhancement does not increase tumor growth rate or metastasis in rabbit VX2 tumors, suggesting that USMC is a safe method to increase tumor perfusion (62).

In groups exposed to low acoustic pressures (0.5, 0.8 and 1.0 MPa), only the 1.0 MPa group exhibited an increase in tumor pH after the USMC treatment. To further elucidate the mechanism by which USMC mitigates TME acidosis, we analyzed the glycolytic metabolism of the tumors. A reduction in hypoxia-inducible factor 1-alpha (HIF-1α) levels after USMC treatment at 1.0 MPa suggests an alleviation of tumor hypoxia. Correspondingly, this treatment also led to a decrease in LDHA and lactic acid levels, indicative of a reduction in glycolytic activity. As tumor growth surpasses the capacity of blood vessel supply, hypoxia intensifies, prompting cancer cells to upregulate HIF-1α (33, 63). This, in turn, increases the expression of key enzymes involved in converting glucose to lactate, such as LDHA (64, 65), which converts pyruvate to lactate, and the lactate extruding monocarboxylate transporter 4 and 1 (MCT4/1) (5), which regulate lactate influx and efflux. Meanwhile, HIF1α inhibits the entry of pyruvate into the TCA cycle by upregulating pyruvate dehydrogenase kinase 1 (PDK1), shifting cellular energy production towards glycolysis (66). Therefore, the reduction of tumor acidosis following USMC treatment is mainly due to the ultrasonic cavitation of microbubbles inducing microvascular dilation and tumor blood perfusion enhancement. This then alleviates tumor hypoxia and subsequently diminishes the glycolytic activity in tumor cells, leading to a decrease in lactic acid production and release.

Current treatments targeting tumor lactic acid, including MCTs inhibitors and LDHA blockade, both show promise in suppressing tumor growth (5, 67, 68). AZD3965, a dual MCT1/2 inhibitor, exhibits significant anti-tumor effects in mouse models and is undergoing clinical trials (69). However, the lactate shuttle in the brain (that is, neuronal MCT2-dependent uptake of lactate released by astrocytes through MCT4 and by oligodendrocytes via MCT1) could limit its use (70, 71). Additionally, systemic buffering with sodium bicarbonate therapy has also been proposed to neutralize tumor acidity and improve responses to treatments like immune checkpoint blockade (ICBs) (10, 72). Despite potential benefits, systemic buffer therapy poses risks such as hypokalemia and QT interval prolongation (73). In contrast, USMC can safely and reversibly reduce tumor acidity without impacting the overall tumor macrostructure.

Tumor pH levels remained unchanged in the 1.5 MPa group, despite a significant decrease in tumor blood perfusion after USMC 1.5 MPa was observed. A possible mechanism is that high-pressure amplitude modifies the tumor ECS, indirectly influencing tumor acidity. As noted before, High-amplitude ultrasound irradiation increases vessel permeability and induces necrosis and apoptosis of cancer cells (74, 75). This may lead to the tumor ECS expanding and facilitate the efflux of micro- and macromolecular substances from the blood vessels, reducing acidic metabolic byproduct accumulation.

Dense ECM in tumor tissues compresses neovasculature, decreases hydraulic conductivity, and also impedes the removal of acidic waste (76). In pancreatic ductal adenocarcinoma (PDAC), collagen-I rich ECM exacerbates the physiological interstitial acidosis and PDAC progression (77). Zhao (78) discovered that low-intensity pulsed ultrasound treatment can reverse myocardial fibrosis caused by prolonged hypoxia via the TRAAK-mediated HIF-1α/DNMT3a signaling pathway. However, our Masson staining showed no significant differences in the collagen area, suggesting that the acoustic parameters we used did not markedly alter the tumor fibrillar collagen macrostructure. Lee (79) proposed that exposure to relatively low power pulse- High intensity focused ultrasound (with peak acoustic powers between 5 and 20 W/cm²) can modify tumor ECM structure, disrupting collagen organization and reducing collagen levels in ECM-rich A549 tumors. In our study, the maximal acoustic pressure reached 1.5 MPa with a central frequency of 1MHz. The ultrasound intensity used in our study (spatial Peak Temporal Average Intensity I(spta) <774mW/cm2) was much lower. Although no observed differences in fibrillar collagen area, potential direct effects on collagen microstructure cannot be discounted. As observed by Hancock (80), the fibril network is deformed into parallel bundles and larger intrafibrillar spaces after applying pulsed ultrasound. Further studies are required to clarify the impacts of USMC on ECM microstructure, such as ECM pore size and fiber alignment, to understand its role in reducing tumor acidic waste accumulation.

Investigations into USMC combined with other treatments also show potential. USMC can dilate targeted tumor vasculature, and may expand extracellular space, which provides a non-invasive and efficient pathway for drug delivery to the targeted tumor site (81). For example, ultrasound combined with patient-derived microvesicles loaded with sonosensitizers displayed superior tumor targeting ability (82). Combining USMC with anti-acids may not only enhance drug accumulation at the tumor site but also achieve a synergistic acid-reducing effect. Furthermore, combining USMC with tumor immunotherapy, such as ICB, is also promising. This combination may modify the acidic and hostile TME, reduce the immunosuppressive milieu caused by tumor acidosis, and facilitate the delivery of immunotherapeutic agents, potentially enhancing the effector function of antitumor immune cells.

Currently, most USMC trials are conducted on animals. While there is substantial promise for clinical application, several limitations and challenges need to be addressed. The complexity and heterogeneity of the human environment, along with regional variations in neoplasm characteristics, affect the efficacy of USMC regimens. Therefore, selecting optimal USMC parameters and matching suitable microbubbles are crucial and warrant further investigation. At present, commercially available microbubbles are used primarily for enhanced ultrasound imaging (83). Developing “next generation” microbubbles with higher stability, ease of production, and preservation is essential for future clinical USMC applications.

A limitation of this study is that the USMC treatment and correlation analysis were only investigated in one tumor model (84, 85). Most animal research employs ultrasound to irradiate solid subcutaneous tumors in mice. For the larger human body, identifying the optimal ultrasound transducers for some specific tissue types and irradiation modality for treating deep-seated or cavernous organ tumors such as gastrointestinal cancer warrants further investigation. Additionally, we did not investigate the effects of various acoustic parameters on tumor blood perfusion and tumor acidity. Parameters such as ultrasound frequency, intensity, and irradiation time require further detailed examination.

In summary, we have shown that tumor acidity correlated well with AUC and PI, indicating that tumor blood perfusion is closely linked to tumor acidity and CEUS can effectively assess acidity in preclinical tumor model. Furthermore, the tumor blood perfusion enhancement induced by USMC 1.0Mpa can reduce tumor glycolysis metabolism and thereby alleviate tumor acidosis. Our study presents a novel methodology for mapping and targeting tumor acidosis.

## Data availability statement

The original contributions presented in the study are included in the article/supplementary material, further inquiries can be directed to the corresponding author/s.

## Ethics statement

The animal study was approved by Laboratory Animal Ethics Committee of the South China University of Technology. The study was conducted in accordance with the local legislation and institutional requirements.

## Author contributions

DQ: Conceptualization, Formal analysis, Investigation, Methodology, Writing – original draft, Data curation. YH: Conceptualization, Data curation, Formal analysis, Investigation, Methodology, Writing – original draft. YF: Conceptualization, Formal analysis, Investigation, Methodology, Writing – original draft, Data curation. ML: Data curation, Software, Writing – original draft. ZL: Data curation, Software, Writing – original draft. ZZ: Methodology, Resources, Writing – original draft. YX: Resources, Software, Writing – review & editing. ZH: Supervision, Writing – review & editing. SM: Supervision, Writing – review & editing. HJ: Supervision, Writing – review & editing. JL: Conceptualization, Funding acquisition, Project administration, Resources, Supervision, Validation, Writing – review & editing.
